# Sex Differences in Early Programming by Maternal High Fat Diet Induced-Obesity and Fish Oil Supplementation in Mice

**DOI:** 10.3390/nu13113703

**Published:** 2021-10-21

**Authors:** Latha Ramalingam, Kalhara R. Menikdiwela, Stephani Spainhour, Tochi Eboh, Naima Moustaid-Moussa

**Affiliations:** 1 Department of Nutritional Sciences and Obesity Research Institute, Texas Tech University, Lubbock, TX 74909, USA; lramalin@syr.edu (L.R.); kalhara.menikdiwela@ttu.edu (K.R.M.); stehphani.spainhour@ttu.edu (S.S.); tochi.eboh@ttuhsc.edu (T.E.); 2 Department of Nutrition and Food Studies, Syracuse University, Syracuse, NY 13244, USA

**Keywords:** maternal obesity, high fat diet, fish oil, sex differences, mice

## Abstract

Pre-pregnancy obesity is a contributing factor for impairments in offspring metabolic health. Interventional strategies during pregnancy are a potential approach to alleviate and/or prevent obesity and obesity related metabolic alterations in the offspring. Fish oil (FO), rich in omega-3 polyunsaturated fatty acids (*n*-3 PUFAs) exerts metabolic health benefits. However, the role of FO in early life remains still unknown. Hence, this study objective was to determine the effect of FO supplementation in mice from pre-pregnancy through lactation, and to study the post-natal metabolic health effects in gonadal fat and liver of offspring fed high fat (HF) diet with or without FO. Female C57BL6J mice aged 4–5 weeks were fed a HF (45% fat) diet supplemented with or without FO (30 g/kg of diet) and low fat (LF; 10% fat) pre-pregnancy through lactation. After weaning, offspring (male and female) from HF or FO dams either continued the same diet (HF-HF and FO-FO) or switched to the other diet (HF-FO and FO-HF) for 13 weeks, creating four groups of treatment, and LF-LF was used as a control group. Serum, gonadal fat and liver tissue were collected at termination for metabolic analyses. Offspring of both sexes fed HF with or without fish oil gained (*p* < 0.05) more weight post weaning, compared to LF-LF-fed mice. All the female offspring groups supplemented with FO had reduced body weight compared to the respective male groups. Further, FO-FO supplementation in both sexes (*p* < 0.05) improved glucose clearance and insulin sensitivity compared to HF-HF. All FO-FO fed mice had significantly reduced adipocyte size compared to HF-HF group in both male and females. Inflammation, measured by mRNA levels of monocyte chemoattractant protein 1 *(Mcp1)*, was reduced (*p* < 0.05) with FO supplementation in both sexes in gonadal fat and in the liver. Markers of fatty acid synthesis, fatty acid synthase (*Fasn*) showed no sex specific differences in gonadal fat and liver of mice supplemented with HF. Female mice had lower liver triglycerides than male counterparts. Supplementation of FO in mice improved metabolic health of offspring by lowering markers of lipid synthesis and inflammation.

## 1. Introduction

Obesity prevalence in pregnant women and children, are alarmingly rising in the United States and globally [1,2,3,4]. Whilst lifestyle and diet are known to contribute to obesity, other factors that include genetic and environmental components play a critical role in its development [5,6]. Importantly, pre-pregnancy obesity affects the offspring’s future health by increasing the risk of metabolic disorders [7,8,9]. Both animal and human studies collectively indicate that excess supply of energy in the in-utero environment affects metabolic phenotype in part by excess accumulation of fat in the offspring [9,10].

Research has indicated that bioactive interventions in rodent models of maternal obesity could prevent adverse effects of maternal obesity and improve metabolic health of the offspring. Few bioactive interventions with positive effects in rodents include fish oil (FO), prebiotics and N acetyl cysteine [11,12,13,14]. This study focuses on fish and dietary FO supplements as they are prescribed during pregnancy [15]. FO contains omega 3 polyunsaturated fatty acids (*n*-3 PUFAs) rich in eicosapentaenoic acid (EPA) and docosahexaenoic acid (DHA), which are known to improve metabolic health and prevent obesity [16,17]. This is in part through reducing inflammation in gonadal fat and lowering triglycerides in the liver and serum [16,18,19]. Further, long term beneficial effects of maternal *n*-3 PUFA supplementation were observed in adipose tissue and liver of offspring as shown by our lab and others [11,13,20,21]. However, in most studies, the offspring were weaned on to chow diet [13,22] or continued on same *n*-3 PUFA diet [23]. Furthermore, most studies focused on male offspring or data for male and female offspring were combined [13]. Whilst, beneficial effects were observed in male offspring, sexual dimorphism exists in terms of metabolic homeostasis, specifically in glucose and fat metabolism pathways [24,25]. Further to our knowledge, none of the studies tested the effects of high fat (HF) diet in offspring following a *n*-3 PUFA maternal diet or vice versa. Given that pre-obesity is a concern, testing the implications of FO intervention during the pre-pregnancy and pregnancy period is timely. The hypothesis of the current study is that prior FO supplementation during pre-pregnancy through lactation would improve post-natal metabolic health effects in gonadal fat and liver of male and female offspring supplemented with HF diet with or without FO.

## 2. Materials and Methods

**Figure 1:**
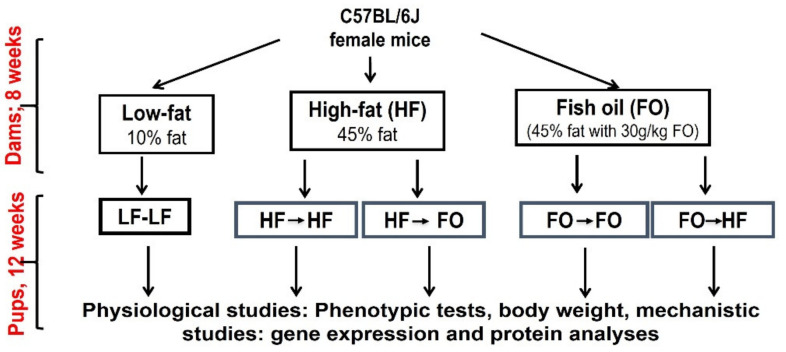
Study design and dietary intervention groups.

**Mice and diets**: All animal protocols were submitted to and approved by the Institutional Animal Care and Use Committee of Texas Tech University, protocol number 16001-04. Male and female virgin C57BL/6J mice aged 4–5 weeks old were shipped from Jackson Laboratory (Bar Harbor, ME, USA). After one-week acclimatization, female mice were fed either a low fat (LF; 20, 70, and 10% of energy from protein, carbohydrate, and fat; n = 12), high-fat (HF; 20, 32, 48% of energy from protein, carbohydrates, and fat; n = 24) or HF supplemented with menhaden oil (n = 24; Omega Proteins, Houston, TX, USA) 30 g/kg diet (FO) for eight weeks. EPA and DHA were ~14% and 11% of the total fatty acids, respectively. Micronutrients were same across all diet groups. Detailed diet composition is provided in Table S1 and the study design is described in Figure 1.

At the end of the eight weeks, female mice were bred with a male mouse fed a chow diet. Female dams were maintained on the same diet during gestation, lactation until weaning (21 days after the birth of offspring). Upon weaning, male and female pups born to LF were maintained on LF and used as a control (LF-LF). Pups born to HF fed dams were randomly divided and either continued on HF (HF-HF) or switched to FO diet (HF-FO). Similarly, pups born to dams fed FO continued on FO (FO-FO) or were switched to HF diet (FO-HF) creating four treatment groups and one control group (LF-LF) (Figure 1). All the mice offspring groups had a minimum of 13–15 replicates which were from different dams as we used 1–2 litter for each group from a single dam. For the gene expression studies, we had n = 8–10 mice and n = 4/group for fatty acid analyses and staining experiments. The lower number for gene expression studies is due to degradation of RNA of few samples. Due to aggression, male offspring were housed individually whereas female mice were grouped during the study period. Mice had access to the respective diets and water ad libitum. The mice were housed in ventilated cages with corn cob bedding at 22 °C and 70% humidity for the duration of the study (13 weeks). All the animal rooms were maintained on 12-h day/night with light onset at 7:00 am. Offspring mice were weighed weekly post weaning and food intake was measured. Glucose and insulin tolerance tests (GTT & ITT, respectively) were performed on offspring in the 10th and 12th weeks of dietary intervention post weaning. Body fat composition was measured using an Echo-MRI analyzer (EchoMRI LLC, Houston, TX, USA) at 11th week of dietary intervention. At the end of dietary intervention (13 weeks), mice were fasted for 5 h before euthanasia. Mice were sacrificed using CO_2_ inhalation, then blood, gonadal fat and liver tissue were harvested. Tissues were snap-frozen in liquid nitrogen and then transferred to −80 °C until further analyses.

**GTT and ITT:** Mice were fasted around 8.00 and GTT and ITT were performed at 13.00. After a 5 h fast, tail blood was drawn for baseline measurement using a glucometer (Abbott Laboratories, Alameda, CA, USA). Mice were then given intraperitoneally 2 g glucose/kg of body weight. Mice were injected with variable volume of 20% glucose based on the body weight. Glucose was measured at 30, 60, 90, and 120-min intervals. For ITT, mice were similarly fasted for 5 h. After the 5 h fast, mice were injected with 1 IU/kg body weight insulin (Humulin; Abbott, Chicago, IL, USA) intraperitoneally. Blood glucose was measured at 15, 30, 60, and 90-min intervals. The area under the curve (AUC) of GTT was assessed and calculated according to the trapezoidal method as we have previously reported [26].

**Immunohistochemical staining**: Upon sacrifice, gonadal fat and liver tissue were parafilm embedded and stained with haematoxylin-eosin (H&E). Digital images were taken under a 20× magnification on an EVOS^®^ FL Auto imaging system (Thermo Fisher Scientific, Waltham, MA, USA). Quantification of the adipocyte area was calculated for a minimum of 100 cells using a validated microanalyzer [27].

**RNA Isolation and quantitative PCR:** RNA was isolated from gonadal fat and liver using RNeasy isolation kit (Qiagen, Germantown, MD, USA) following manufacturer protocol. Total RNA was then reverse transcribed into cDNA using the maxima reverse transcription kit (Thermo Fisher, Carlsbad, CA, USA), which was used for performing quantitative PCR of various target genes (sequences provided in Table S2), normalized to TATA-Box Binding Protein (*Tbp* as control). We used delta delta CT method for relative quantification of gene expression.

**Fatty Acid analyses:** Fatty acids were extracted from red blood cells (RBC) from the liver and gonadal fat [28]. The analysis was conducted using thermo trace gas chromatography—ISQ quadrupole MS GC equipment with TR-FAME at Texas Tech Chemistry Core Facility.

**Serum Measurement and Liver Triglycerides:** Serum levels of resistin, insulin, leptin, and adiponectin were measured using a MILLIPLEX MAP Mouse Adipocyte Magnetic Panel (MADKMAG-71K) in luminex multiplexing system (Luminex xMAP, Millipore, Darmstadt, Germany). Liver triglycerides were measured using a colorimetric assay kit, 10010303 (Cayman Chemical, Ann Arbor, MI, USA).

**Statistical analyses:** One-way analysis of variance (ANOVA) was used for detecting diet specific differences within one sex, followed by Tukey’s posthoc test in GraphPad Prism (version 8). We performed 2-way ANOVA followed by Sidak post hoc test to identify the interaction between diet and sex which is provided in Table 1. Results are presented as mean ± SEM (standard error of mean), with statistical significance considered at *p* < 0.05.

## 3. Results

### 3.1. Metabolic Alterations with FO in Male and Female Offspring

**Figure 2:**
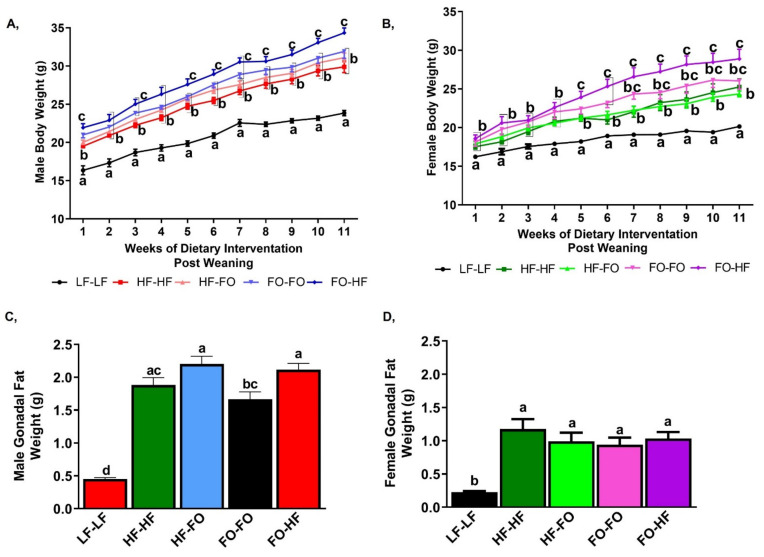
**Fish oil effects on post weaning body weight in male and female mice: **(**A**,**B**) Weekly body weight measurements post weaning in (**A**) male offspring, (**B**) female offspring. (**C**,**D**) Mean gonadal fat weight measured during sacrifice in (**C**) male mice (**D**) female mice. Data is presented as mean ± SEM (n = 10–13). Statistical differences are determined for each time point for line graphs. Common letters indicate no differences across the groups at each specific time point. Common letters on the error bars indicate no significance (e.g., “a” is significantly different from “b” and “ab” indicates no significance compared to “a” and “b”).

We observed that the pre-pregnancy weights of HF and FO dams were significantly different from LF fed dams, but there was no difference between the HF and FO dams (Figure S1A). HF feeding increased body weight at all weeks in both male and female offspring compared to LF-LF fed groups (Figure 2A,B). Male offspring born to HF or FO dams had comparable body weights post weaning (Figure 2A). Interestingly, at most time points, FO-HF offspring had higher body weight than HF-HF offspring (Figure 2A). Food intake was lower in all the groups supplemented with HF compared to LF-LF (Figure S1B). However, no difference was observed in food intake between any of the groups supplemented with HF or FO. Further, HF diet had higher energy content at 4.85 Kcal/g vs. the LF-LF at 3.85 Kcal/g. Hence despite lower food intake, the HF group had significantly higher energy intake. HF feeding did not alter body weight in female mice until four weeks of post weaning dietary intervention. FO-FO fed female offspring had higher body weight only at weeks six and seven, compared to HF-HF group (Figure 2B). Consistent with males, FO-HF female mice had higher body weight compared to HF-HF, with no differences observed between FO-FO and FO-HF offspring (Figure 2B). HF feeding increased final body weight in male and female offspring compared to LF-LF mice, with no differences between groups fed HF or FO at end of experiments (Figure S1C,D). Female mice fed HF with FO (HF-FO, FO-FO and FO-HF) at any time point had reduced body weight compared to respective male groups (Table 1).

HF feeding increased gonadal fat weight in male and female groups compared to LF-LF mice (Figure 2C,D). Furthermore, gonadal fat weight was comparable between HF-HF and HF-FO male groups. FO-HF group had increased gonadal weight compared to FO-FO male offspring (Figure 2C). HF or FO fed female offspring had comparable gonadal fat weights (Figure 2D). Furthermore, an interaction was observed between sex and diet for gonadal fat weight.

Based on the interaction, HF or FO supplementation (HF-FO, FO-FO and FO-HF) in male mice had significantly higher gonadal fat weight compared to female groups, with no difference between male and female LF-LF groups (Figure 2C,D), suggesting higher fat pad weight in males fed HF.

**Figure 3:**
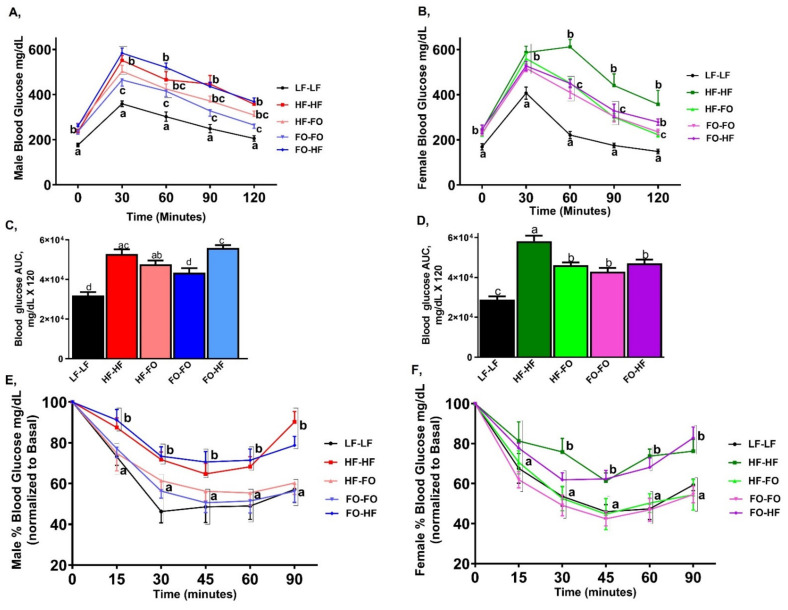
**Fish oil effects on glucose clearance in male and female mice:**(**A**,**B**) Glucose tolerance test (GTT) in male and female mice. (**C**,**D**) Area under the curve for GTT in male and female mice. (**E**,**F**) Insulin tolerance test (ITT) in male and female mice. Data is presented as mean ± SEM (n = 13–15). Common letters on the error bars indicate no significance (e.g., “a” is significantly different from “b” and “ab” indicates no significance compared to “a” and “b”). Statistical differences are determined for each time point for line graphs. Common letters indicate no differences across the groups at each specific time point.

We evaluated glucose tolerance by GTT. HF feeding significantly increased basal glucose levels in male and female offspring compared to LF-LF group (Figure 3A,B). HF-HF & HF-FO male groups had similar glucose clearance. FO-HF male offspring had higher glucose intolerance compared to FO-FO group, indicated by a higher area under the curve (AUC) as shown in Figure 3C. Furthermore, FO-FO supplementation lowered glucose intolerance in male and female offspring compared to HF-HF offspring (Figure 3B,D). For AUC, no sex differences were observed, although interaction between diet and sex was found. For ITT, FO-FO and HF-FO groups showed improved insulin sensitivity similar to LF-LF groups for both male and female mice, and most importantly FO-FO and HF-FO and LF-LF groups were clearly separated from HF fed groups (HF-HF and FO-HF) from 30-min time point onwards (Figure 3E,F).

### 3.2. Effects of Fish Oil Supplementation on Adipose Markers in Males and Females

**Figure 4:**
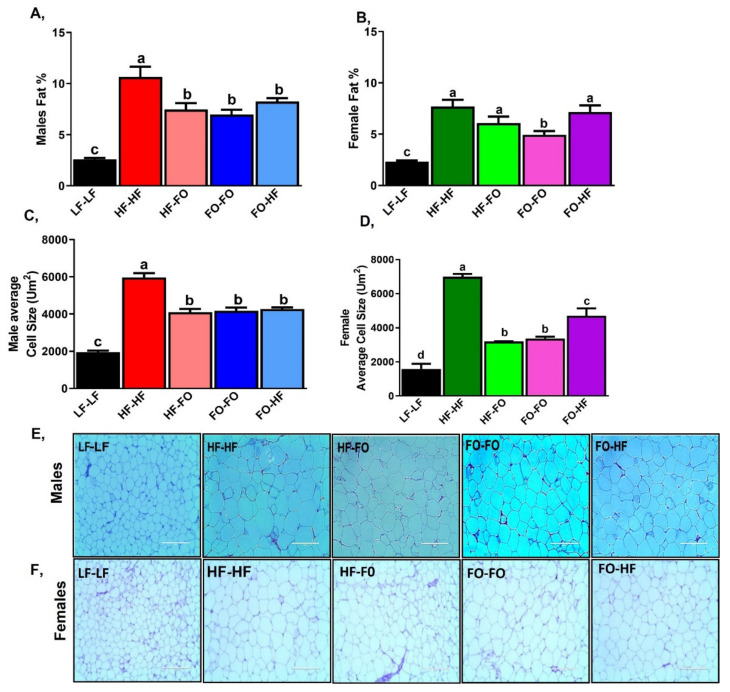
**Fish oil supplementation alters overall adiposity in both male and female mice**:(**A**,**B**) Body fat composition in male and female mice. (**C**–**F**) Mean adipocyte cell area in male and female mice along with their representative images (20x magnification and 200µm scale). Data is presented as mean ± SEM (n = 4). Common letters on the error bars indicate no significance (e.g., “a” is significantly different from “b” and “ab” indicates no significance compared to “a” and “b”).

HF-HF fed male and female offspring had higher body fat percentages compared to LF-LF mice (Figure 4A,B). Furthermore, FO feeding during either utero or development reduced body fat percentage compared to HF-HF male offspring (Figure 4A). HF-FO female offspring showed comparable body fat percentage to HF-HF female offspring, while FO-HF females had significantly higher body fat percentage compared to FO-FO offspring (Figure 4B). Only HF-HF and FO-FO male mice had higher body fat percentage than the respective female offspring groups based on 2-way ANOVA (Table 1). No interaction between diet and sex was found for body fat percentage (Table 1).

In terms of adipocyte area, HF feeding increased adipocyte area in male and female offspring compared to LF-LF mice, as shown in Figure 4C–F. FO supplementation either during in utero environment or development in males reduced adipocyte area compared to HF-HF (Figure 4C). HF-FO female offspring had lower adipocyte size compared to HF-HF offspring, while FO-HF had higher adipocyte area compared to FO-FO offspring (Figure 4D). Adipocyte size between respective male and female HF fed groups was comparable based on 2-way ANOVA (Table 1), but an interaction between diet and sex was found specifically for the female LF-LF group having lower adipocyte area compared to the LF-LF male group (Figure 4E,F).

**Figure 5:**
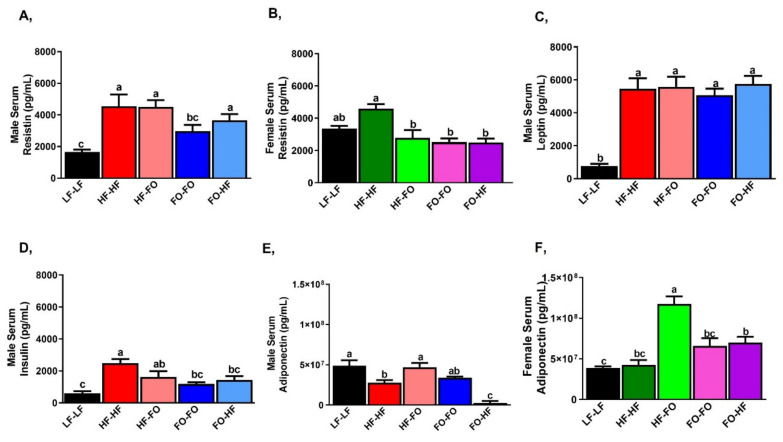
**Fish oil supplementation alters adipokines in serum of male and female mice:** (**A**) Serum resistin levels in male mice. (**B**) Serum resistin levels in female mice. (**C**) Serum leptin in male mice (**D**) Serum insulin levels in male mice. (**E**,**F**) Serum adiponectin levels in both males and females. Data is presented as mean ± SEM (n = 8). Common letters on the error bars indicate no significance (e.g., “a” is significantly different from “b” and “ab” indicates no significance compared to “a” and “b”).

HF feeding increased serum resistin levels in male offspring compared to LF-LF (Figure 5A). FO-FO supplementation lowered resistin serum levels compared to HF-HF offspring (Figure 5A). In females, serum resistin levels were not different among LF-LF and HF-HF fed offspring (Figure 5B). However, FO supplementation during gestation or development (HF-FO, FO-FO and FO-HF) significantly lowered resistin levels compared to HF-HF female offspring (Figure 5B). In comparing males versus females by 2-way ANOVA, LF-LF resistin levels were higher in females, while FO-HF female offspring had lower resistin levels than respective male offspring group. Serum leptin levels were higher in HF group than male offspring fed LF-LF (Figure 5C). HF or FO feeding did not alter leptin levels. Similar changes in leptin levels were observed for female mice (Figure S1E), however female groups that were fed FO (HF-FO, FO-FO and FO-HF) had lower leptin levels compared to the respective male offspring based on 2-way ANOVA (Table 1).

HF-HF feeding increased serum insulin levels in male and female offspring compared to LF-LF group (Figure 5D and Figure S1F). HF feeding did not alter insulin level among male or female offspring born to HF. Similarly, FO feeding did not alter insulin levels among male or female offspring born to HF. However, FO supplementation during gestation (FO-FO & FO-HF) reduced insulin levels in male and female offspring compared to respective HF-HF offspring. Only HF-HF and HF-FO female offspring had lower insulin levels than the respective male groups based on 2-way ANOVA.

HF feeding lowered serum adiponectin levels compared to LF-LF fed males (Figure 5E). HF-FO group had increased serum adiponectin levels compared to HF-HF male offspring. Further, FO-HF offspring had reduced adiponectin levels compared to FO-FO male offspring (Figure 5E). Surprisingly, only HF-FO female offspring had higher adiponectin levels compared to other groups (Figure 5F). All FO supplemented female offspring groups (HF-FO, FO-FO and FO-HF) had significantly higher adiponectin levels than male offspring based on 2-way ANOVA.

**Figure 6:**
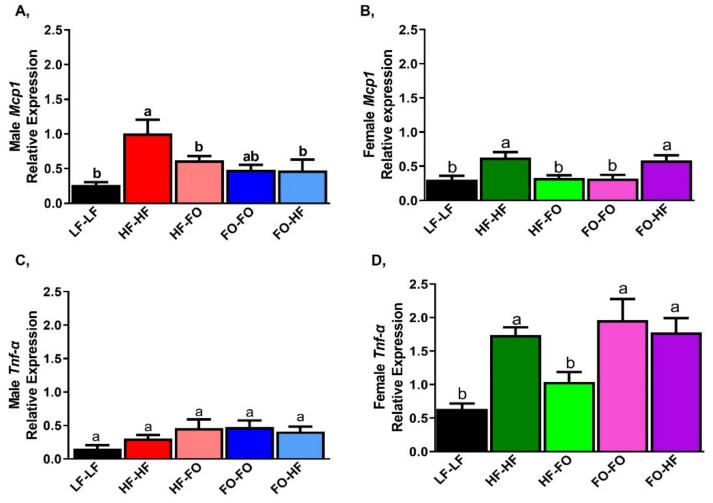
**Fish oil supplementation alters pro-inflammatory markers in mice gonadal fat.** (**A**,**B**) mRNA levels of pro-inflammatory marker monocyte chemoattractant protein (*Mcp1*) in male and female mice. (**C**,**D**) mRNA levels of pro-inflammatory marker tumor necrosis factor alpha (*Tnf alpha*) in male and female mice. Data is presented as mean ± SEM (n = 8). Common letters on the error bars indicate no significance (e.g., “a” is significantly different from “b” and “ab” indicates no significance compared to “a” and “b”).

HF-HF feeding increased mRNA levels of monocyte chemoattractant protein-1 (*Mcp1)* in male and female offspring compared to their respective LF-LF offspring (Figure 6A,B). FO supplementation either during gestation or development reduced *Mcp1* mRNA levels compared to HF-HF in males (Figure 6A). HF-FO and FO-FO female offspring had lower *Mcp1* mRNA levels compared to HF-HF (Figure 6B). Moreover, FO-HF female offspring had higher *Mcp1* mRNA levels compared to FO-FO group. As expected, HF-HF female offspring had significantly lower *Mcp1* mRNA levels than male HF-HF offspring group based on 2-way ANOVA. Similar changes were observed for adipose interleukin 6 (*Il-6)* mRNA levels as shown in Figure S2A,B. No differences were observed for tumor necrosis factor alpha *(Tnf-α)* mRNA levels across male mice (Figure 6C). HF-FO female offspring had lower *Tnf-α* levels compared to HF-HF (Figure 6D).

Interestingly, FO or HF supplementation increased *Tnf-α* mRNA levels in females compared to male offspring based on 2-way ANOVA (Table 1).

**Figure 7:**
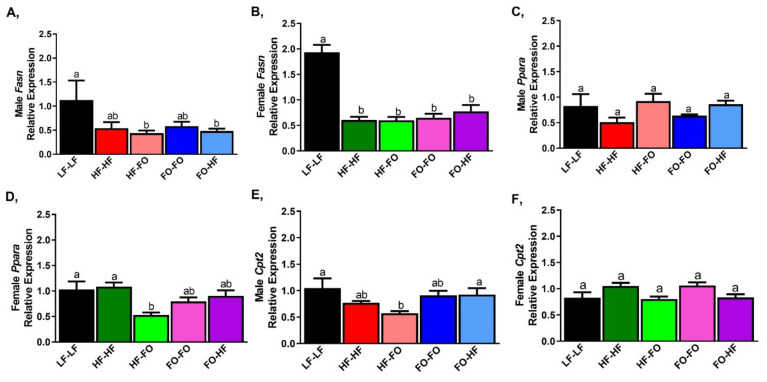
**Fish oil alters markers of lipid metabolism in mice gonadal fat.** (**A**,**B**) mRNA levels of fatty acid synthase (*Fasn*) in gonadal fat of male and female mice. (**C**,**D**) mRNA levels of fatty acid oxidation markers peroxisome proliferator activated receptor alpha (*Ppara*) in gonadal fat of male and female mice. (**E**,**F**) mRNA levels of carnityl palmitoyl transferase-2 (*Cpt2*) in gonadal fat of male and female mice. Data is presented as mean ± SEM (n = 8 all groups). Common letters on the error bars indicate no significance (e.g., “a” is significantly different from “b” and “ab” indicates no significance compared to “a” and “b”).

Lipid synthesis was measured with markers that include fatty acid synthase (*Fasn*) and acetyl-CoA carboxylase (*Acaca*). mRNA levels of *Fasn* were lower (trending towards significance for *Fasn*) in HF-HF male (Figure 7A) and female offspring (Figure 7B) compared to LF-LF offspring. However, no differences were observed in any of the groups that received HF or FO either during gestation or early development in male and female mice. Similar changes were observed for adipose *Acaca* mRNA levels as shown in Figure S2C,D. Based on sex and diet interaction, only LF-LF female mice had significantly higher *Fasn* mRNA levels than the male mice based on 2-way ANOVA.

Fatty acid oxidation measured by peroxisome proliferator-activated receptor alpha (*Ppara*) did not differ between any male offspring groups (Figure 7C). In female offspring, only HF-FO offspring had lower levels of *Ppara* compared to HF-HF and other groups (Figure 7D). Only the HF-HF male group has lower Ppara compared to the female group, based on sex versus diet interaction. Carnityl palmitoyl transferase-2 (*Cpt2*), another fatty acid oxidative marker, was lower only in HF-FO male offspring group compared to LF-LF but not different in the HF or FO supplemented groups (Figure 7E). No differences in *Cpt2* were observed in females (Figure 7F).

### 3.3. FO Reduced Triglycerides in the Liver in Part by Reducing Inflammation and Lipid Metabolism

**Figure 8:**
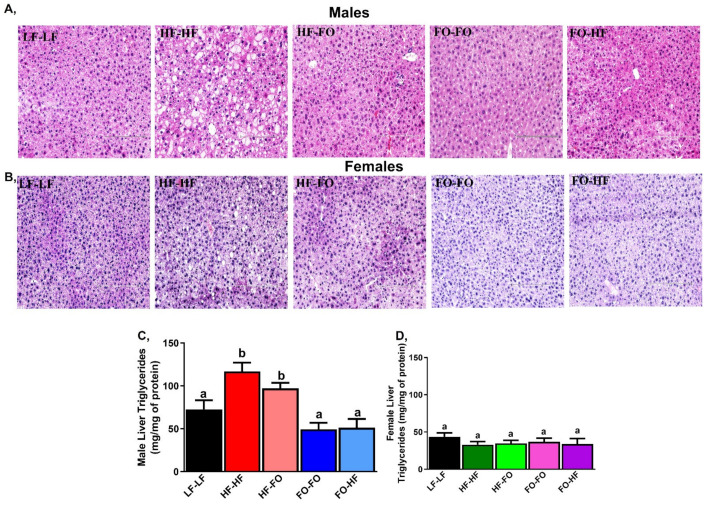
**Fish oil role in liver in males and females.** (**A**,**B**) Haematoxylin and eosin (H&E) staining images for liver from male and female mice (20x magnification and 200 µm scale) (**C**,**D**) Liver triglycerides measured by calorimetry was lower in males and females. Data is presented as mean ± SEM (n = 4 for H&E staining and n = 8–13 liver triglycerides). Common letters on the error bars indicate no significance (e.g., “a” is significantly different from “b” and “ab” indicates no significance compared to “a” and “b”).

As shown in Figure 8A, HF-HF mice had higher triglyceride (Tg) accumulation shown by increased white droplets. However, FO supplemented groups during early life or development had lower Tg accumulation levels in male offspring. However, in the female offspring, no difference was observed across groups in Figure 8B. In line with this, liver Tg levels were measured by colorimetric assays. HF-HF offspring had higher liver Tg levels compared to LF-LF fed male offspring (Figure 8C). FO-FO & FO-HF male offspring had lower Tg levels compared to HF-FO & HF-HF with no differences between offspring born to HF or FO groups. All female groups had comparable levels of Tg levels (Figure 8D). All female offspring mice had reduced Tg levels in comparison to male offspring based on 2-way ANOVA (Table 1).

**Figure 9:**
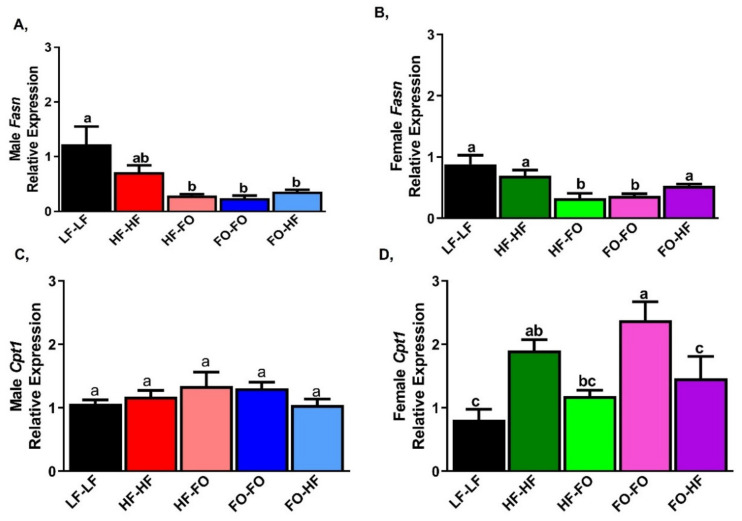
**Fish oil alters markers of lipid metabolism in liver of males and females.** (**A**,**B**) mRNA levels of fatty acid synthase (*Fasn*) in liver of males and female mice. (**C**,**D**) mRNA levels of carnityl palmitoyl transferase-2 (*Cpt2*) in liver of male and female mice. Data is presented as mean ± SEM (n = 8). Common letters on the error bars indicate no significance (e.g., “a” is significantly different from “b” and “ab” indicates no significance compared to “a” and “b”).

In the liver, there were no differences in mRNA levels of *Fasn* between HF-HF and LF-LF fed male and female offspring. HF or FO supplemented male groups had comparable mRNA levels of *Fasn* (Figure 9A,B). HF-FO female offspring had lower *Fasn* mRNA levels compared to HF-HF group (Figure 9B). FO-HF female offspring had increased *Fasn* mRNA levels compared to FO-FO groups (Figure 9B). HF fed female offspring groups and LF-LF had significantly lower mRNA levels of *Fasn* than those of the respective male offspring groups. No interaction was found between diet and sex for *Fasn*. mRNA levels of *Acaca* were measured and data were similar to *Fasn* mRNA levels (Figure S3A,B).

Male offspring groups had comparable mRNA levels of beta oxidation marker, *Cpt1*, (Figure 9C). In HF-HF females, *Cpt1* mRNA levels were increased in comparison with LF-LF. However, there were no differences between HF-HF and HF-FO. Further, FO-HF group had lower mRNA level of *Cpt1* compared to FO-FO female group (Figure 9D). Based on diet versus sex interaction, Only FO-FO female offspring was significantly different from male offspring group.

**Figure 10:**
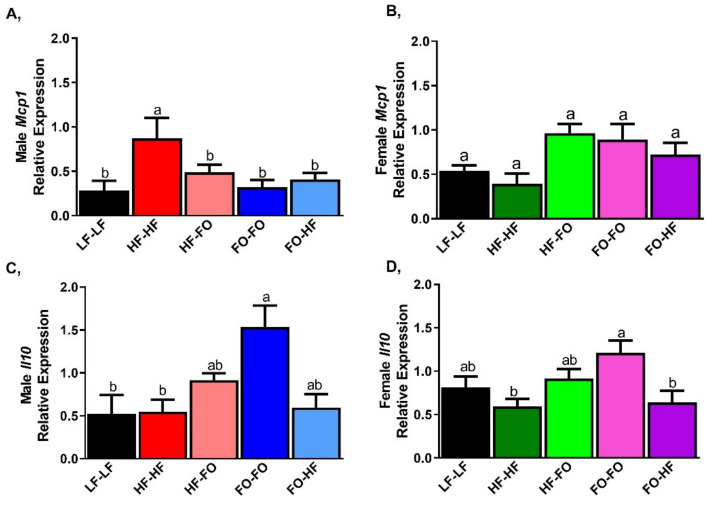
Fish oil supplementation reduces pro-inflammatory markers in Liver of mice: (**A**,**B**) mRNA levels of pro-inflammatory marker monocyte chemoattractant protein (*Mcp1*) in liver of male and female mice. (**C**,**D**) mRNA levels of interleukin 10 (*Il10*) in male and female mice. Data is presented as mean ± SEM (n = 8). Common letters on the error bars indicate no significance (e.g., “a” is significantly different from “b” and “ab” indicates no significance compared to “a” and “b”).

As observed in adipose tissue, HF feeding increased *Mcp1* mRNA levels in male offspring compared to LF-LF (Figure 10A). FO supplementation during gestation or development lowered *Mcp1* mRNA levels in male offspring. In females, HF and LF-LF fed offspring had no difference in *Mcp1* mRNA levels (Figure 10B). Interestingly, *Mcp1* was trending higher in groups that were supplemented with FO compared to HF-HF offspring in females (Figure 10B). Based on 2-way ANOVA, female HF-FO and FO-HF groups had higher *Mcp1* mRNA levels compared to male offspring mice. Further no sex differences were found for *Il6* gene levels. Interleukin 10 (*Il10*) mRNA levels were not different between LF-LF and HF fed male and female offspring (Figure 10C,D). Further, HF-HF & HF-FO male and female offspring had comparable *Il10* mRNA levels. Male FO-FO & FO-HF offspring had no difference in *Il10* mRNA levels, but FO-FO female offspring had higher *Il10* mRNA levels compared to HF-HF (Figure 10C,D).

**Figure 11:**
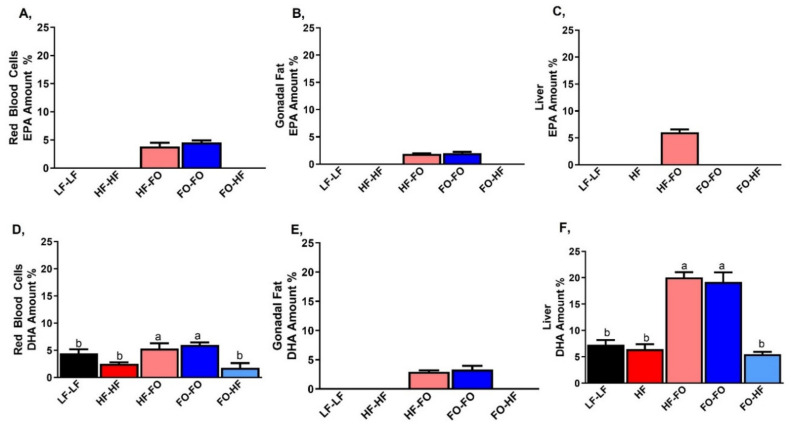
Incorporation of omega-3 fatty acids in serum and tissues: Amount of eicosapentaenoic acid (EPA) incorporation into (**A**) Blood (**B**) White adipose tissue (gonadal fat) and (**C**) Liver. Similarly, amount of docosahexaenoic acid (DHA) into (**D**) Blood (**E**) gonadal fat and (**F**) Liver. Data is presented as mean ± SEM (n = 4). Common letters on the error bars indicate no significance (e.g., “a” is significantly different from “b” and “ab” indicates no significance compared to “a” and “b”).

Lastly, we also confirmed that the EPA and DHA in the diet reached tissues (gonadal fat, liver, and blood) in male offspring groups. EPA was detectable only in groups that were supplemented with FO as shown in Figure 11A–C. In the red blood cells, EPA was 4% of the total fatty acids, while lower amounts were detected in adipose tissue at 2% of the total fatty acids. Similarly, higher DHA amount was detectable in groups supplemented with FO, with the highest in liver, followed by blood and adipose as shown in Figure 11D–F. Saturated fatty acid content was also measured but was unaltered between the diets in red blood cells, gonadal fat and liver, as shown in Tables S3–S5.

## 4. Discussion

It is known that interventions such as lifestyle, diet and physical activity during pregnancy are beneficial in early programming in the offspring [25,29]. However, it is unclear if nutritional interventions during early life is beneficial for the offspring. This study identified that FO supplementation during pregnancy was beneficial, even when offspring were challenged with HF. To our knowledge, this is the first study to identify that supplementation of FO during early life had beneficial properties and is independent of the offspring diet in part by reducing inflammation in gonadal fat and liver. Further, our study established that sex-specific differences were observed with FO in terms of lipid metabolism and inflammation in the liver and adipose tissue during early life programming, which is the strength of this paper.

We measured resistin, which is a marker for insulin resistance and observed lower resistin levels in female offspring supplemented with FO either during pregnancy or early life compared to male offspring. The lower resistin levels are likely because female offspring have better mechanisms to cope with HF diet and are less susceptible to the HF diet challenge as shown in other studies [30]. Further, for adiponectin, our data is in line with studies in humans, where women have higher adiponectin levels which contributes to better insulin sensitivity than their male counterparts [31]. While it is known such differences exist, the mechanisms are not entirely understood.

We focused on gonadal fat as FO is known to lower inflammation predominantly in the gonadal adipose tissue depot. Interestingly, for *Il6* gene expression, we saw comparable effects between males and females. While, for *Mcp1* and *Tnf-α*, differences were observed between sexes, indicating different pathways were altered in males versus females in response to FO. This could be because metabolites of EPA and DHA known as specialized pro-resolving mediators (SPM) are higher in female mice compared to male mice [32].

High fat feeding in the offspring following FO supplementation during the intrauterine window exacerbated adverse effects of HF on serum markers and glucose tolerance test. However, FO supplementation during early development prevented increases in fatty acid synthesis and inflammation in adipose tissue after HF feeding with stronger effect in males. Most of the markers measured were higher in males fed HF or FO compared to respective groups in female mice. This is because C57BL/6J male mice, the strain used in this study respond better to HF diet compared to females [33]. This is in line with studies where pancreatic islets from female mice cope functionally better than males in response to intrauterine HF [34,35].

Dysregulated inflammation, along with disrupted adipose metabolism are critical underlying features of obesity [36]. Due to dysregulated adipocyte metabolism and impaired hepatic de novo lipid metabolism, free fatty acid efflux from adipose tissue are potential contributors to non-alcoholic fatty liver disease (NAFLD) [37]. Increased secretion of inflammatory markers in adipose tissue increases inflammatory markers in the liver, as shown previously by our lab and others [18,19]. We saw increases in serum adiponectin levels and altered inflammatory signals with FO supplementation, even though we did not observe changes in final body weight in males and females. This is line with studies that show alteration in inflammation but no changes in body weight [38]. Surprisingly, we did not see differences in lipogenic genes in terms of fatty acid synthesis or oxidation in adipose tissue but saw beneficial effects of FO in the liver. In earlier studies from our lab, we observed altered markers of lipid metabolism in adipose and in the liver in mice fed with EPA enriched FO [39]. We probably did not see differences in markers of lipid metabolism since we used equal amount of EPA and DHA compared to other studies where we used EPA enriched FO. This is in line with studies which have demonstrated distinct responses to EPA and DHA at least in the liver [40]. Other possibility is that these markers could be altered by post translational modifications.

The composition of the fatty acids in the diet regulates metabolism differently. While a HF diet rich in saturated fatty acids increases adiposity [41], PUFAs have anti-obesity effects, in part, by reducing triglycerides and inflammation [19,42]. We measured fatty acid composition in blood, gonadal fat and liver. The results we observed is due to incorporation of PUFAs in the tissues and not due to lowering the amounts of saturated fatty acids (SFA) as we did not observe difference in SFA content across groups (Tables S3–S5). Even though we observed higher amount of EPA in the liver, we observed similar alterations in adipose and liver supporting that lower amount of FO is sufficient to reproduce these effects. This is line with studies that our lab and others recently published in terms of similar beneficial metabolic effects even with lower doses of EPA enriched FO [43,44]. We used a combination of DHA and EPA (~30 g of FO/diet) which has ~10 g of FO; this is higher than the current recommendation of 4 g/day for people suffering from hypertriglyceridemia [45].

We used FO (30 g FO/kg diet) based on previous studies in our lab. This translates to ~10 g per day in humans. Mice consumed on average 20 g of food per week. Based on that, they were exposed ~ to 85–90 mg of FO per day. We have not identified any detrimental effects of the doses we used in our diet induced obese mouse models, while 10 g is considered on the higher end and over recommended dose, several clinical studies, aimed at reducing hypertriglyceridemia used 4–6 g per day of pharmaceutical grade pure FO [46,47,48,49].

We acknowledge an important limitation of this study is that we did not analyze the effects of FO under LF fed conditions. We tested anti-inflammatory effects of FO under diet induced obese conditions and will perform similar studies in the future using LF diets with and without FO. Further, even though we used littermates of male and female mice, we housed the female mice together at optimal density, while male mice were housed individually which could contribute to some nutritional confounding factor especially energy expenditure. However, we did not measure energy expenditure in this study. But the mice were all littermates, on the same genetic background. We housed the males individually due to their aggressive behaviour. Lastly, we fed mice FO during the entire gestation period. It would be interesting in the future to find optimal periods of nutritional intervention during the development period. In conclusion, we found sexual dimorphism in the role of FO in protecting against obesity in offspring, partly by reducing inflammation which is independent of the offspring diet.

## Figures and Tables

**Table 1:** Diet and sex interaction across groups (2-Way ANOVA).

	Sex	Diet	Sex Vs Diet Interaction
Body weight	<0.0001	<0.0001	0.358
GTT AUC	0.179	<0.0001	0.021
Epididymal weight	<0.0001	<0.0001	0.0008
Fat Percentage	<0.0001	<0.0001	0.093
Adipocyte Size	0.347	<0.0001	0.0003
Serum			
Resistin	0.220	<0.0001	0.001
Leptin	<0.0001	<0.0001	0.0704
Insulin	<0.0001	<0.0001	0.0212
Adiponectin	<0.0001	<0.0001	<0.0001
White Adipose Tissue Gene Expression			
*Mcp-1*	0.024	<0.0001	0.035
*Il-6*	0.523	<0.0001	0.001
*Tnf-α*	<0.0001	<0.0001	0.001
*Fasn*	0.003	<0.0001	0.022
*Acaca*	<0.0001	<0.0001	<0.0001
*Ppara*	0.116	0.289	0.003
*Cpt 2*	0.210	0.004	0.030
Liver Data			
Liver Triglycerides	<0.0001	<0.004	<0.0001
*Fasn*	0.947	<0.0001	0.087
*Acaca*	<0.0001	<0.0001	0.959
*Cpt 1*	0.012	0.001	0.007
*Mcp-1*	0.004	0.102	0.003
*Il-6*	0.904	0.001	0.652
*Il-10*	0.915	<0.0001	0.472

All 5 groups (LF, HF-HF, HF-FO, FO-FO and FO-HF) of male and female mice were compared within each other. A *p* < 0.05 for dietary interaction indicates that at least one dietary group was significantly different from another group.

## Supplementary Materials

The following are available online at https://www.mdpi.com/article/10.3390/nu13113703/s1. Figure S1: Body weight and serum parameters of offspring mice, Figure S2: Levels of Pro-inflammatory and fatty acid metabolism markers in adipose tissue of offspring mice, Figure S3: Levels of Pro-inflammatory and fatty acid metabolism markers in liver. Table S1: Diet Composition. Table S2: Primer sequences used in the study. Table S3: Fatty Acid analyses of Blood. Table S4: Fatty Acid analyses of White Adipose Tissue. Table S5: Fatty Acid analyses of Liver.
