# Supplementary material for: Sex Differences in Early Programming by Maternal High Fat Diet Induced-Obesity and Fish Oil Supplementation in Mice
Source: Nutrients. 2021 Oct 21;13(11):3703. doi: 10.3390/nu13113703 (PMC8625698; doi:10.3390/nu13113703)
Supplement: Supplementary file 1 [file nutrients-13-03703-s001.zip › nutrients-1409994-supplementary.pdf]

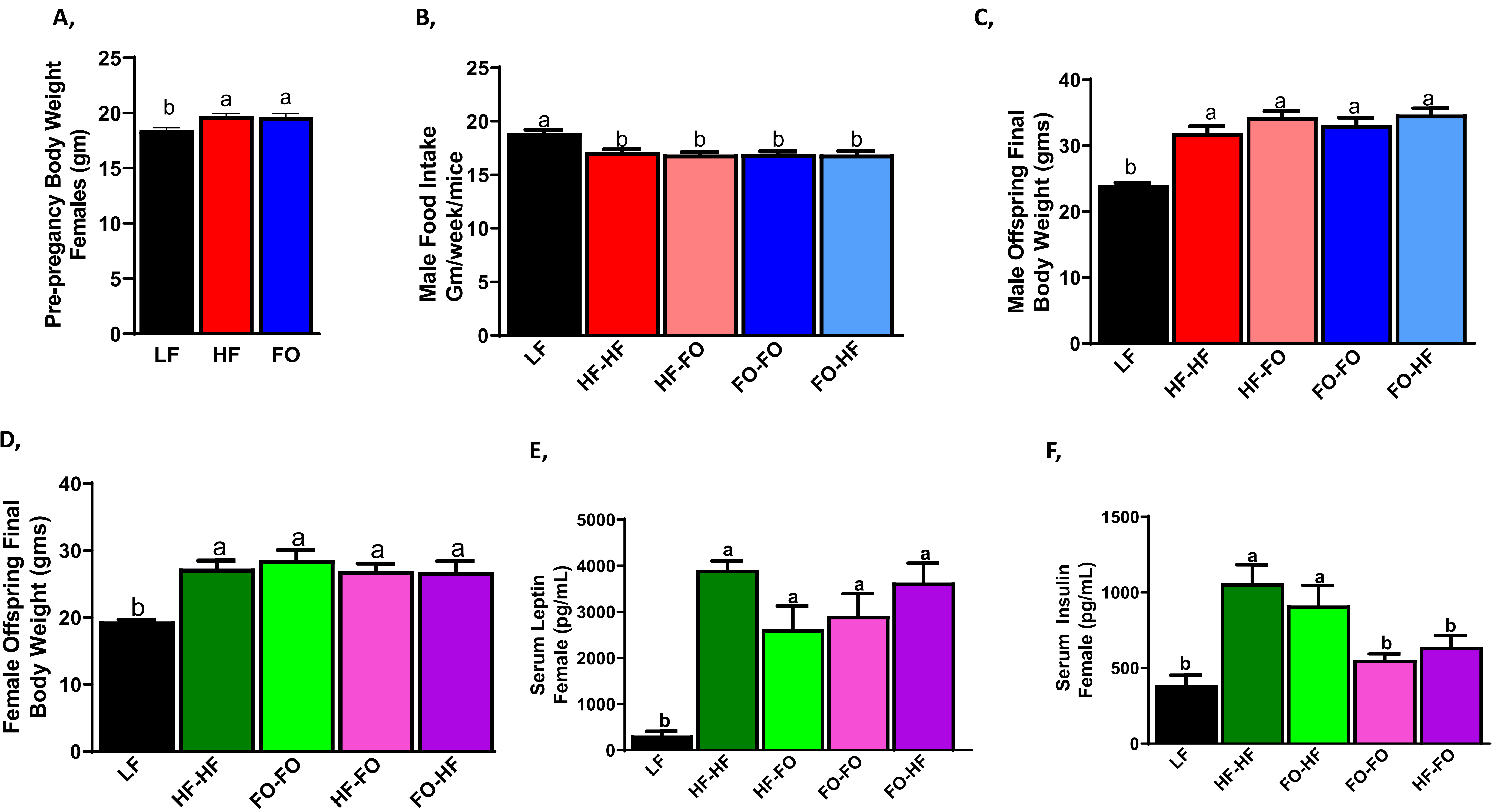

Figure 1. (A) Pre-pregnancy weight of female mice after eight weeks of dietary intervention. (B) Average food in per week in male offspring mice post weaning (C) Final body weight of male mice at sacrifice (D) Final body weight of male mice at sacrifice (E) Serum leptin levels in female mice (F) Serum insulin levels in female mice. Data is presented as mean ± SEM (n=8). Common letters on the error bars indicate no significance (e.g., “a” is significantly different from “b” and “ab” indicates no significance compared to “a” and “b”).

Supplementary Figure S2

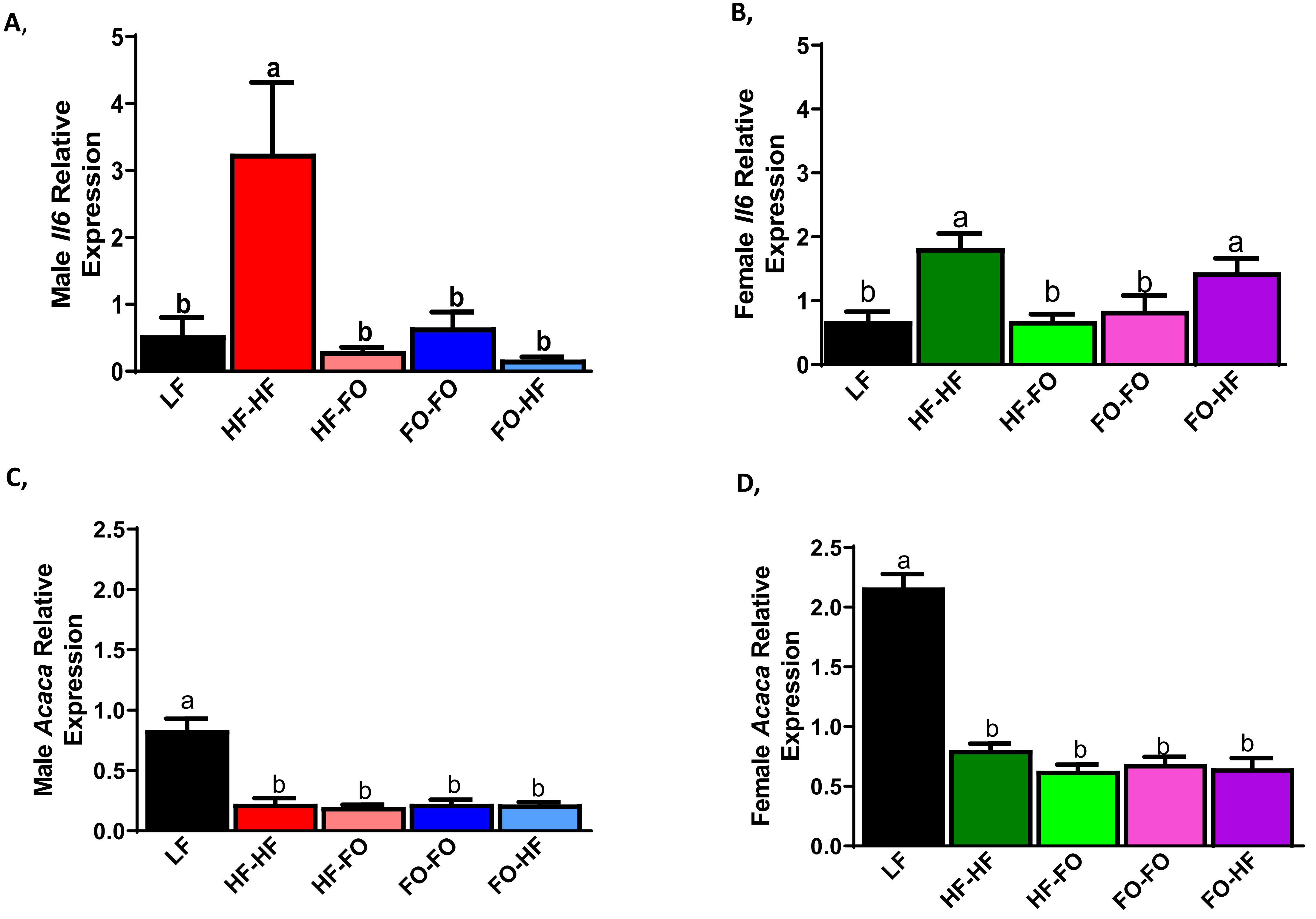

Figure 2. (A-B) mRNA levels of pro-inflammatory marker interleukin -6 (Il6) in gonadal fat of male and female mice. (C-D) mRNA levels of fatty acid synthesis marker acetyl co A carboxylase (Acaca) in in gonadal fat of male and female mice. Data is presented as mean ± SEM (n=8). Common letters on the error bars indicate no significance (e.g., “a” is significantly different from “b” and “ab” indicates no significance compared to “a” and “b”).

Supplementary Figure S3

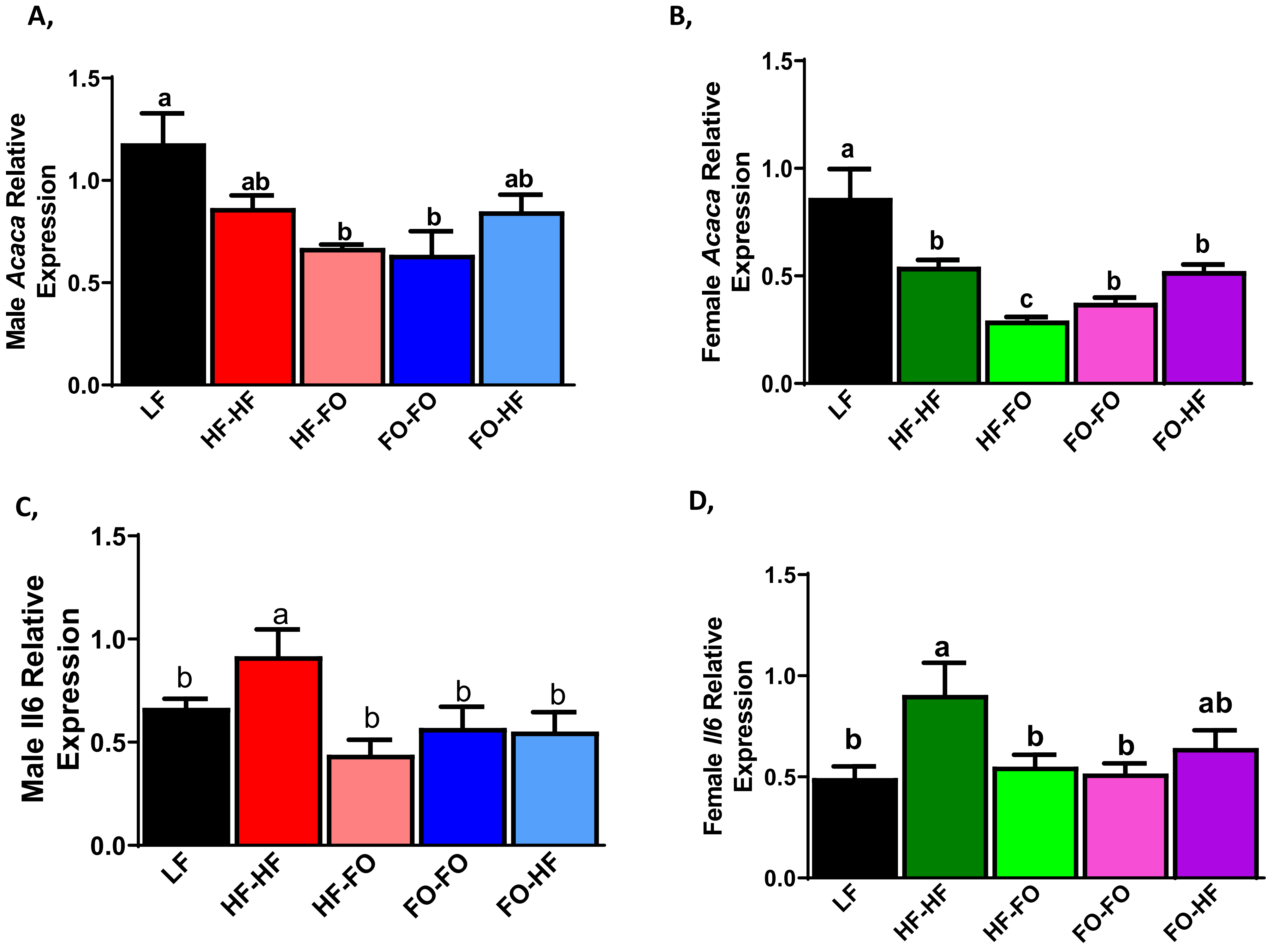

Figure 3. (A-B) mRNA levels of fatty acid synthesis marker acetyl co A carboxylase (Acaca) in liver of male and female mice. (C-D) mRNA levels of pro-inflammatory marker interleukin -6 (Il6) in liver of male and female mice. Data is presented as mean ± SEM (n=4). Common letters on the error bars indicate no significance (e.g., “a” is significantly different from “b” and “ab” indicates no significance compared to “a” and “b”).

Supplemental Table S1: Diet Composition

	Low Fat D15062103		High Fat-Fish oil D15062101		High Fat D15062102	
	g	*kcal%*	g	*kcal%*	g	*kcal%*
Protein	19.2	*20*	24.2	*20*	24.2	*20*
Carbohydrate	67.3	*70*	38.2	*32*	38.2	*32*
Fat	4.3	*10*	26	*48*	26	*48*
Total		*100*		*100*		*100*
kcal/g	3.85		4.84		4.84	
**Ingredient**	**g**	***kcal***	**g**	***kcal***	**g**	***kcal***
Casein, 30 mesh	200	*800*	200	*800*	200	*800*
L-Cystine	3	*12*	3	*12*	3	*12*
Corn Starch	427	*1708*	38	*152*	38	*152*
Maltodextrin 10	100	*400*	100	*400*	100	*400*
Sucrose	173	*691*	173	*691*	173	*691*
Cellulose	50	*0*	50	*0*	50	*0*
Soybean Oil	25	*225*	25	*225*	25	*225*
Lard	20	*180*	144	*1296*	193	*1737*
Menhaden Oil, Nutegrity	0	*0*	49	*441*	0	*0*
Mineral Mix, S10026	10	*0*	10	*0*	10	*0*
DiCalcium Phosphate	13	*0*	13	*0*	13	*0*
Calcium Carbonate	5.5	*0*	5.5	*0*	5.5	*0*
Potassium Citrate, 1 H2O	16.5	*0*	16.5	*0*	16.5	*0*
Vitamin Mix, V10001	10	*40*	10	*40*	10	*40*
Choline Bitartrate	2	*0*	2	*0*	2	*0*
Vitamin E Acetate, 50% (500 IU/g)	0.13	*0*	0.13	*0*	0.13	*0*
**Total**	**1055**	***4057***	**839**	***4057***	**839**	***4057***

Supplemental Table S2: Primer sequences used in the study

Primer	Forward sequence	Reverse Sequence
Mcp-1	ACTTCTATGCCTCCTGCTCAT	GCTGCTTGTGATTCTCCTGTAG
Tnf α	TCTCAAAACT CGAGTGACAAGC	GGTTGTCTTTGAGA TCCATGC
Fasn	GTCGTCTATACCACTGCTTACT	ACACCACCTGAACCTGAG
Ppara	TCGAGGAAGGCACTACACCT	TCTTCCCAAAGCTCCTTCAA
Cpt2	CAGCACAGCATCGTACCCA	TCCCAATGCCGTTCTCAAAAT
Cpt1	GAGACAGACACCATCCAACAC	GAGCCAGACCTTGAAGTAACG
Il-6	AACCGCTATGAAGTTCCTCTC	TCCTCTGTGAAGTCTCCTCTC
Il-10	TCTTACTGACTGGCATGAGGAT	GCATTAAGGAGTCGGTTAGCA
Tbp	CAGCCTTCCA CCTTATGCTC	CGTAAGGCATCATTGG ACT

Supplemental Table S3: Fatty Acid analyses of Blood

	LF-LF	HF-HF	HF-FO	FO-FO	FO-HF	P-value
PUFA						
Linoleic acid	8.67 ± 0.47	9.77 ± 0.40	7.87 ± 1.35	7.07 ± 1.93	8.48 ± 0.81	NS
Eicosapentaenoic acid	0.00 ± 0.00 [b]	0.00 ± 0.00 [b]	3.83 ± 0.67 [a]	4.57 ± 0.36 [a]	0.00 ± 0.00 [b]	< 0.0001
cis-11,14-Eicosadienoic	0.00 ± 0.00	0.15 ± 0.15	0.24 ± 0.24	0.15 ± 0.06	0.00 ± 0.00	NS
cis-8,11,14-Eicosatrienoic	0.83 ± 0.11 [ab]	0.95 ± 0.18 [a]	0.32 ± 0.14 [ab]	0.39 ± 0.13 [ab]	0.21 ± 0.21 [b]	< 0.01
Docosahexaenoic acid	3.25 ± 0.48 [acb]	2.53 ± 0.28 [ac]	5.32 ± 0.98 [ab]	6.01 ± 0.45 [b]	1.81 ± 0.84 [c]	< 0.003
MUFA						
Palmitoleic acid	2.75 ± 0.61 [a]	0.35 ± 0.20 [bc]	1.42 ± 0.33 [ab]	1.12 ± 0.19 [bc]	0.00 ± 0.00 [c]	< 0.0001
cis-9-Oleic acid	16.2 ± 1.36	13.2 ± 0.52	12.1 ± 2.06	12.8 ± 0.84	11.3 ± 1.27	NS
SFA						
Tetradecanoic	0.93 ± 0.22 [a]	0.13 ± 0.13 [b]	1.15 ± 0.17 [a]	1.12 ± 0.20 [a]	0.00 ± 0.00[b]	< 0.0001
Palmitic acid	31.7 ± 1.25	27.8 ± 1.18	28.5 ± 4.28	31.2 ± 0.62	26.0 ± 1.68	NS
Stearic acid	10.7 ± 0.76	14.2 ± 0.63	11.8 ± 2.15	13.7 ± 1.20	13.4 ± 1.02	NS
Tricosanoic	14.7 ± 1.41 [a]	15.1 ± 1.17 [a]	5.61 ± 1.06 [b]	6.32 ± 0.63 [b]	13.3 ± 1.01 [a]	< 0.0001
UFA: trans-9-Elaidic	10.4 ± 3.34	15.7 ± 2.82	21.7 ± 1.01	15.2 ± 4.19	25.5 ± 6.06	NS

[1]Values are means ± SEMs, *n* = 4. Means without a common superscript letter are significantly different, $P < 0.05$. FO, fish oil; HF, high fat; LF-LF, low-fat control diet.

Supplemental Table S4: Fatty Acid analyses of White Adipose Tissue

	HF-HF	HF-FO	FO-FO	FO-HF	LF-LF	P-value
PUFA						
Linoleic acid	0.00 ± 0.00	0.00 ± 0.00	0.00 ± 0.00	0.00 ± 0.00	0.00 ± 0.00	NS
Eicosapentaenoic acid	0.00 ± 0.00	1.86 ± 0.14	2.01 ± 0.26	0.00 ± 0.00	0.00 ± 0.00	< 0.0001
cis-11,14-Eicosadienoic acid	0.66 ± 0.04 ^{a}	0.67 ± 0.05 ^{a}	0.63 ± 0.08 ^{a}	0.89 ± 0.13 ^{a}	0.26 ± 0.09 ^{b}	<0.05
cis-8,11,14-Eicosatrienoic acid	0.00 ± 0.00	0.08 ± 0.09	0.00 ± 0.00	0.00 ± 0.00	0.00 ± 0.00	NS
Docosahexaenoic acid	0.00 ± 0.00	2.95 ± 0.21	3.32 ± 0.63	0.00 ± 0.00	0.00 ± 0.00	< 0.0026
MUFA						
Palmitoleic Acid	7.77 ± 0.35 ^{a}	10.9 ± 0.38 ^{a}	12.5 ± 0.94 ^{a}	10.4 ± 0.96 ^{a}	18.6 ± 2.18 ^{b}	< 0.0001
cis-9-Oleic Acid	39.3 ± 0.54^{a}	27.0 ± 2.32 ^{ab}	23.9 ± 2.94 ^{b}	31.5 ± 6.45 ^{ab}	31.5 ± 0.88 ^{ab}	<0.05
SFA						
Tetradecanoic acid	0.81 ± 0.05^{a}	2.48 ± 0.12 ^{a}	2.97 ± 0.33 ^{a}	1.21 ± 0.09 ^{a}	1.71 ± 0.14 ^{a}	NS
Palmitic acid	11.0 ± 0.09 ^{abc}	14.0 ± 0.50 ^{b}	15.7 ± 0.73 ^{ab}	6.14 ± 3.54 ^{c}	13.5 ± 0.59 ^{ba}	< 0.01
Stearic acid	0.71 ± 0.71 ^{a}	3.69 ± 0.25 ^{b}	2.89 ± 0.99 ^{ab}	3.66 ± 0.22 ^{b}	1.50 ± 0.51 ^{ab}	NS
Tricosanoic	0.54 ± 0.03 ^{a}	0.53 ±0.03 ^{a}	0.55 ± 0.04 ^{a}	0.71 ± 0.04 ^{b}	0.47 ± 0.01 ^{a}	< 0.001
UFA: trans-9-Elaidic	4.57 ± 1.42	0.39 ± 0.07	2.16 ± 1.99	0.08 ± 0.08	0.00 ± 0.00	NS

[1]Values are means ± SEMs, $n = 4$. Means without a common superscript letter are significantly different, $P < 0.05$. FO, fish oil; HF, high fat; LF-LF, low-fat control diet.

Supplemental Table S5: Fatty Acid analyses of Liver

	LF-LF	HF-HF	HF-FO	FO-FO	FO-HF	P-value
PUFA						
Eicosapentaenoic acid	0.00 ± 0.00	0.00 ± 0.00	6.02 ± 0.537	0.00 ± 0.00	0.00 ± 0.00	< 0.05
Docosahexaenoic acid	7.30 ± 0.87	6.44 ± 0.96	20.0 ± 0.994	19.2 ± 1.81	5.50 ± 0.42	< 0.05
Linoleic acid	14.8 ± 1.58 [a]	19.7±1.39 [b]	15.9 ± 0.53 [a]	13.0 ± 0.48[a]	16.4 ± 1.07 [a]	< 0.05
cis-11,14-Eicosadienoic acid	0.30 ± 0.10	0.29 ± 0.10	0.00 ± 0.00	0.00 ± 0.00	0.00 ± 0.00	NS
cis-8,11,14-Eicosatrienoic acid	1.40 ± 0.25	0.94 ± 0.03	0.73 ± 0.03	0.81 ± 0.02	1.01 ± 0.07	NS
MUFA						
Palmitoleic acid						< 0.001
cis-9-Oleic acid	29.7 ± 2.02 [a]	27.2 ± 3.17 [a]	19.6 ± 3.76 [b]	19.6 ± 2.44 [b]	31.9 ± 1.58 [ac]	< 0.05
SFA						
Tetradecanoic	0.74 ± 0.26	0.36 ± 0.13	0.45 ± 0.02	0.47 ± 0.05	0.62 ± 0.05	NS
Palmitic acid	22.2 ± 0.99	23.2 ± 1.32	22.3 ± 1.13	24.6 ± 1.26	25.1 ± 1.04	NS
Stearic acid	6.87 ± 0.61	7.22 ± 1.30	6.79 ± 1.46	6.76 ± 0.62	6.54 ± 0.67	NS
Tricosanoic	10.7 ± 1.97 [a]	10.7 ± 1.98 [a]	4.98 ± 1.19 [b]	5.34 ± 0.90 [b]	8.76 ± 0.75 [a]	< 0.05
UFA: trans-9-Elaidic	0.00 ± 0.00	0.00 ± 0.00	0.00 ± 0.00	0.00 ± 0.00	0.00 ± 0.00	NS

[1]Values are represented as % of total fatty acids and are means ± SEMs, *n* = 4. Means without a common superscript letter are significantly different, *P* < 0.05. FO, fish oil; HF, high fat; LF-LF, low-fat control diet.
